# Amazonian Guarana- and Açai-Conjugated Extracts Improve Scratched Fibroblast Healing and *Eisenia fetida* Surgical Tail Amputation by Modulating Oxidative Metabolism

**DOI:** 10.1155/2022/3094362

**Published:** 2022-06-26

**Authors:** Fellipe D. Felin, Ednea A. Maia-Ribeiro, Carollina D. Felin, Nathália A. C. Bonotto, Bárbara O. Turra, Isabel Roggia, Verônica F. Azzolin, Cibele F. Teixeira, Moisés H. Mastella, Carolina Rodrigues de Freitas, Jaqueline Greijanim, Daniel Santos, Erico M. M. Flores, Fernanda Barbisan, Ivana B. M. Cruz, Tiango A. Ribeiro

**Affiliations:** ^1^Mestrado Profissional em Ciências da Saúde, Universidade Federal de Santa Maria (UFSM), Santa Maria, RS, Brazil 97105-9000; ^2^Universidade do Estado do Amazonas-Fundação Universidade Aberta da Terceira Idade (FUNATI), Manaus, AM, Brazil 69029-000; ^3^Pontíficia Universidade Católica do Rio Grande do Sul, Porto Alegre, RS, Brazil 90619-900; ^4^Programa de Pós-Graduação em Gerontologia, Universidade Federal de Santa Maria (UFSM), Santa Maria, RS, Brazil 97105-9000; ^5^Programa de Pós-Graduação em Farmacologia, Universidade Federal de Santa Maria (UFSM), Santa Maria, RS, Brazil 97105-9000; ^6^Laboratório de Biogenômica, Universidade Federal de Santa Maria (UFSM), Santa Maria, RS, Brazil 97105-9000; ^7^Departamento de Química, Universidade Federal de Santa Maria (UFSM), Santa Maria, RS, Brazil 97105-9000; ^8^Departamento de Patologia, Universidade Federal de Santa Maria (UFSM), Santa Maria, RS, Brazil 97105-9000

## Abstract

**Background:**

Previous studies have suggested that guarana (*Paullinia cupana*) and açai (*Euterpe oleracea*) have antioxidant, anti-inflammatory, and proliferative properties, indicating their potential therapeutic action in wound healing. We produced a conjugated guarana-açai (GA) extract and tested its healing action on earthworms (*Eisenia fetida*) subjected to tail amputation by surgical incision.

**Methods:**

Extract from roasted guarana seeds and fresh açai seed berries was produced. The antioxidant and genoprotective capacity of GA extract was tested. The concentration with the most remarkable healing potential was used in subsequent tests. The last three posterior segments of the clitellate earthworm tail reared under standardized conditions were surgically amputated. Next, topical PBS or GA extract was applied to the surgical wound. The rate of cell migration and tissue regeneration at the local wound site was histologically evaluated after the procedure. Expression of the SOX4 gene that acts in epithelial-to-mesenchymal transition was determined by RT-qPCR.

**Results:**

Sixteen bioactive molecules, including some previously described substances, were identified. All tested concentrations exhibited antioxidant and genoprotective effects. The GA extract accelerated the healing processes as observed through macroscopic and histological analyses and increased expression of SOX4.

**Conclusion:**

The GA extract has a potential role in the healing of surgically induced wounds.

## 1. Introduction

There is a clear correlation involving demographic population aging and an increase in the frequency of plastic surgery, since people are living longer and are more concerned with maintaining a more youthful appearance, personal autonomy, and health. Although aesthetic surgical procedures are typically elective and usually performed on healthy patients, some undesirable postsurgical complications can occur including infections, local anaesthetic systemic toxicity, electrolyte and hematologic alterations, intravascular fluid shifts, and wound healing abnormalities [1]. Therefore, optimal management of postoperative wounds is important to prevent potential complications such as the appearance of thick, deformed scars and keloids and development of nodules under the skin, which is caused by the formation of hard tissue [2].

One factor that seems to mitigate the occurrence of complications in surgical healing is patient nutritional status. Evidence has shown that malnutrition or deficiency in certain nutrients and bioactive molecules present in some foods can cause impairment of the complex biological and molecular events of the wound healing process. For example, deficiency can alter postsurgical events such as coagulation, inflammation, migration, proliferation, and skin remodeling [3]. In addition to oral ingestion, current studies have suggested that the topical application of certain extracts or drugs based on bioactive molecules from fruits and other herbal plants could have beneficial effects on wound healing processes [4–8].

Populations living in the Amazon rainforest use herbal plants and products obtained from certain fruits to relieve various clinical symptoms such as inflammation, pain, and fever and for wound healing. This is the case for guarana (*Paullinia cupana*) seed powder and açai (*Euterpe oleracea*), which have important antioxidant and anti-inflammatory properties [9–15]. Studies have shown that these fruits have an effect on the reversal of mitochondrial dysfunctional states and antiaging properties [16–19]. An investigation performed by [20] also reported that interactions between low-level laser therapy and guaraná extract present antioxidant, anti-inflammatory, and antiapoptotic effects and promote fibroblast proliferation.

These previous results support the hypothesis that a conjugated extract based on roasted guarana seeds and fresh açai berry seeds (GA) could have an effect on the healing processes resulting after acute trauma, such as surgical procedures. To test this hypothesis, we produced an aqueous GA-conjugated extract in which the main bioactive molecules were identified. After safety tests using keratinocyte and fibroblast cell lines, *in vitro* and *in vivo* assays were conducted using human fibroblasts and surgical amputation of the tails of the Californian red earthworm *Eisenia fetida*. The role of oxidative metabolism modulation in healing processes in these experimental models was also determined.

## 2. Materials and Methods

### 2.1. Chemicals and Equipment

All chemicals used in this study were purchased from the following companies: Gibco® Life Technologies Inc. (Grand Island, NY, USA), Vitrocell-Embriolife (Campinas, São Paulo, Brazil), Qiagen (Hilden, North Rhine-Westphalia, Germany), Ludwig Biotechnology (Alvorada, Rio Grande do Sul, Brazil), Bio-Rad Laboratories (Hercules, CA, USA), and Sigma-Aldrich Co. (St. Louis, MO, USA). Molecular biology reagents were as follows: TRIzol reagent (Thermo Fisher Scientific, Grand Island, NY, USA), iScript cDNA synthesis kit (Bio-Rad Laboratories), DNase (Invitrogen Life Technologies; Carlsbad, CA, USA), QuantiFast SYBR Green PCR Kit (Qiagen), and Equipment NanoDrop™ 1000 Spectrophotometer (Thermo Fisher Scientific), thermal cycler, MaxyGene II, Axygen® (Corning Incorporated, Nova York, USA), Rotor-Gene Q 5plex HRM System (Qiagen). The HFF-1 and HaCaT cell lines were obtained from the American Type Culture Collection (ATCC, USA). Protocols involving spectrophotometric and fluorimetric analyses were performed using a 96-well microplate reader (SpectraMax M2/M2e Multimode Plate Reader; Molecular Devices, Sunnyvale, CA, USA), Leica DMI 4000 B microscope (Leica Microsystems GmbH, Wetzlar, Germany), and FACSCanto Flow Cytometer™ II (BD Biosciences, San Jose, California, USA). Use Annexin V-FITC Apoptosis Detection Kit (BD Biosciences, San Jose, California, USA). Data acquisition and cell content analysis were performed using FlowJo vX.0.7 software (Tree Star, Inc., Ashland, OR, USA).

### 2.2. Plant Materials

Toasted guarana powder and fresh açai berries were obtained from agricultural producers in Maués city, Amazonas, and transferred to the laboratory where the extract was produced. The açai berries were pulped, and only the seeds were used to produce the GA-conjugated extract. The use of açai seed is justified because the industrialization of açai pulp generates a large amount of residues that are considered environmental pollutants and which may still have biological properties of interest to human health [21]. The extract was produced only with hot water at a low pH via the addition of citric acid. The GA-conjugated extract was then lyophilized for further analysis of its chemical composition using high-resolution electrospray ionization time-of-flight mass spectrometry (ESI-ToF-MS), as well as *in vitro*, noncellular, and *in vivo* assays.

### 2.3. General Experimental Design

The following experimental protocols were conducted: (1) safety indicators of the GA-conjugated extract at concentrations of 0, 1, 3, 5, 10, and 30 *μ*g/mL to evaluate the effect on the viability and proliferation rate of two human commercial cell lines: keratinocytes (HaCaT) and fibroblasts (HFF-1). These two cell lines are used once keratinocytes and fibroblasts participate actively in the healing process, and our intention was to mimic the wound healing process in the surface scars and usual acute scars. Additionally, both cell lines have been previously used in scratch assay by other authors [22, 23]. The antioxidant and genoprotective capacities of the extracts were determined by 2,2-diphenyl-1-(2,4,6-trinitrophenyl)hydrazyl (DPPH) and GEMO noncellular tests. Overall, these results allowed us to choose the minimal concentration of GA-conjugated extract with adequate safety and efficacy properties. The following protocols were performed with the chosen GA-conjugated extract. (2) The GA-conjugated extract was evaluated *in vitro* on fibroblast cultures through scratched wound healing assays analyzing cultures 3, 6, 24, and 72 h after monolayer physical injury [24]. The modulatory effect of GA extract on oxidative markers, apoptosis, cell cycle, and gene expression associated with fibroblast function was analyzed in 24 h and 72 h cell cultures. (3) *In vivo* assays were performed using *E. fetida* as an experimental model in which the terminal 5 posterior segments of the tail were surgically removed using a scalpel. The wounds were washed with 1 mL of physiological salt solution immediately after cutting and once a day for 3 days. The earthworm tail wound was then exposed to 2 *μ*L of buffer solution (control) or GA-conjugated extract. Cellular migration and scarring events were histologically evaluated 1, 3, 6, 12, and 24 h after the procedure. As SOX4 transcription factor gene is considered an important gene in epithelial-to-mesenchymal transition (EMT) [25], its modulation was compared in injured tissues of earthworms with and without exposure to GA-conjugated extract 3 and 24 h after the surgical tail incision.

### 2.4. Cell Culture and Treatments

*In vitro* investigations used two commercial cell lines, keratinocytes (HaCaT) and a fibroblast cell line (HFF-1), which were cultured in Dulbecco's Modified Eagle's Medium (DMEM) with 10% fetal bovine serum (FBS), supplemented with 1% penicillin/streptomycin and amphotericin B. Cells were maintained at 37°C in a 5% CO_2_ and 95% humidified atmosphere and were expanded by obtaining the optimal amount for experiments. After cell attachment, cells were treated with GA-conjugated extract at concentrations of 1, 3, 5, 10, and 30 *μ*g/mL. After determining the concentration of GA-conjugated extracts, a time curve was plotted over 1, 3, 6, and 24 h to evaluate reactive oxygen species (ROS) production and superoxide anion levels, nitric oxide, and lipoperoxidation. Other parameters were evaluated after 24 and 72 h of incubation.

### 2.5. Scratch Assay In Vitro

At 80% confluence, the fibroblast monolayer cell was streaked in a straight line using a 200 *μ*L sterile pipette, thus simulating an *in vitro* wound. We then used sterile PBS to wash the cells to remove cellular debris. Then, we added the appropriate culture medium and treatment with the conjugated GA extract. Wound photographs were taken at 0, 6, 24, and 72 h to investigate and analyze the scratched wound. The scratched area was measured using ImageJ software. Digital photographs were obtained using a Leica DMI 4000 B microscope.

### 2.6. Cell Viability Assays

Three complementary protocols were used for viability analysis after 24 h of exposure to GA-conjugated extracts: (1) MTT, (2) neutral red, and (3) ATP assays.

#### 2.6.1. MTT

MTT (3-(4,5-dimethyl-2-thiazolyl)-2,5-diphenyl-2H-tetrazolium bromide) assays were performed according to the instructions provided by [26]. Briefly, the supernatants of the treatments were removed, and the cells were resuspended in PBS (0.01 M; pH 7.4). MTT was dissolved to 5 mg/mL in PBS, and 10 *μ*L was added to a 96-well plate containing sample treatments and was subsequently incubated for 1 h at 37°C. The supernatant was removed from the wells, and the cells were resuspended in 200 *μ*L of dimethyl sulfoxide (DMSO). Optical absorbance was measured at 560 nm.

#### 2.6.2. Neutral Red

The neutral red uptake assay provides a quantitative estimate of the number of viable cells in a culture, based on the ability of viable cells to bind and incorporate neutral red dye in lysosomes. Briefly, 100 *μ*L (50 *μ*g/mL) of neutral red dye was added to 96-well cell culture plates. The plates were then incubated for 3 h at 37°C in the dark. The cells were then washed with PBS pH 7.4, and 100 *μ*L of the desorption solution (50% EtOH, 49% H_2_O, 1% glacial acetic acid solution) was added. Optical absorbance was measured at a wavelength of 540 nm [27].

#### 2.6.3. ATP Assay

According to the manufacturer, Promega Corporation, the CellTiter-Glo® cell viability assay detects live cells by quantifying the amount of ATP present in living cells. The reagent quickly lyses the cells, stabilizes the ATP present, and generates a luminescent signal proportional to the amount of ATP present. The signal is directly proportional to the number of living cells in the sample. The test was performed according to the manufacturer's instructions and those contained in the package insert of the kit.

### 2.7. Noncellular Antioxidant and Genoprotective Assays

#### 2.7.1. DPPH Assay

The antioxidant capacity of the GA-conjugated extract was evaluated using 1,1-diphenyl-2-picryl-hydrazyl (DPPH) assays to compare samples with rutin, a pure antioxidant molecule. All tests were performed in triplicate. The antioxidant capacities were described in terms of IC50 (concentration of sample required to scavenge 50% of DPPH free radicals).

#### 2.7.2. Genoprotective–GEMO Assay

Double-stranded DNA (dsDNA) from calf thymus, a reference prooxidant, H_2_O_2_, and a dye specific for dsDNA, PicoGreen, which is part of the Quant-IT™ kit (Thermo Fisher Scientific Waltham, Massachusetts, USA), were used to verify the genoprotective potential of the extracts. PicoGreen DNA. The test assumes that if the investigated compound has a protective capacity, DNA fragmentation will be attenuated and fluorescence of a test group will decrease compared to a positive control group (obtained by exposing calf DNA to H_2_O_2_). The 96-well plate was filled with 10 *μ*L of calf thymus DNA (1 *μ*g/mL plus 70 *μ*L of TE buffer) containing varying concentrations of the GA-conjugated extract and 70 *μ*L of H_2_O_2_ (3 *μ*M). The reaction mixture was then incubated for 30 min. After 30 min, PicoGreen® DNA dye was added, and the fluorescence was read (excitation at 480 nm/emission at 520 nm). The genoprotective effect was considered to be present when the absorbance was lower than that of the positive control group [28].

### 2.8. Apoptosis Detection by Flow Cytometry

Complementary analysis via flow cytometry confirmed that treatment with the GA-conjugated extract was safe. Cytotoxicity was assessed using the Annexin V-FITC Apoptosis Detection Kit, allowing the assessment of early apoptotic cells (annexin V positive, propidium iodide (PI) negative), necrotic cells (annexin V positive, PI positive), and viable cells (annexin V negative, PI negative). The protocol was performed according to BD Biosciences® instructions. Briefly, cells were seeded in 6-well plates at density 1 × 10^6^ cells/mL; after 24 hours, wound “scratching” was carried out. Plates were immediately treated with GA-conjugated extract (5 *μ*g/mL) and incubated for 24 hours. After incubation, cells were trypsinized, washed twice with PBS pH 7.0, and resuspended in 1× binding buffer. The resuspended cells were gently vortexed and stained with 5 *μ*L of annexin-V-FITC and 5 *μ*L of PI as described by kit manufacturer. To avoid induction of apoptosis by mechanical stimulation, the sample was vortexed for more than five seconds. After 15 min incubation in the dark at 22-25°C, 400 *μ*L of 1× binding buffer was added to each tube, and the fluorescence of each cell was analyzed by flow cytometry according to the manufacturer's specifications.

### 2.9. Oxidative Marker Assays

Modulation of oxidative stress was evaluated in cultured cells of the HFF-1 lineage 3, 6, 24, and 72 h after scratching and treatment with GA-conjugated extract (5 *μ*g/mL) by analyzing the levels of superoxide, ROS, nitric oxide, and lipoperoxidation.

#### 2.9.1. Superoxide

Superoxide levels were quantified using a colorimetric assay that produces a formazan salt via reaction with nitroblue tetrazolium chloride (NBT), according to a protocol established by [29].

#### 2.9.2. ROS by 2,7-Dichlorodihydrofluorescein Diacetate

DCFH-DA is a nonfluorescent chemical that is deacetylated by mitochondrial esterase enzymes to DCFH, which reacts with ROS to form DCF, a fluorescent molecule. The assay was performed as described by [30].

#### 2.9.3. Nitric Oxide

Nitric oxide levels were indirectly quantified by analyzing nitrate abundance using the Griess reagent, as described by [31].

#### 2.9.4. Lipoperoxidation

Lipid peroxidation was spectrophotometrically estimated through the formation of thiobarbituric acid reactive substances (TBARS), as previously described by [32].

### 2.10. Earthworm Rearing Conditions and Ethical Issues

Earthworms were commercially obtained and acclimated in the laboratory of 144 Biogenomics, Federal University of Santa Maria, Brazil, for 7 days at 21°C ± 1°C in a Bio-Oxygen Demand (BOD) (Thoth, São Paulo, Brazil) incubator under a 12/12 h dark/light cycle. Before being used in experiments, the earthworms were reared for 30 days in small plastic boxes (18.5 × 18.5 × 6.5 cm) protected from light and containing sterilized soil and cattle manure (10 : 1 proportion) at 80%–85% humidity. The soil used in this treatment was sterilized in an oven at 200°C for 2 h to kill nematodes and other potential worms, parasites, and pathogens. Approximately 500 g of wet soil was placed in each container. The experiments were conducted using juvenile earthworms selected based on the absence of well-developed clitella [33], to avoid possible reproduction-related bias. The experiments were performed independently in triplicate. In many countries, including Brazil, prior approval by an Animal Ethics Committee for studies involving earthworms is not required. However, all experiments were performed in accordance with the ethical principles of animal experimentation that aim to avoid discomfort and unnecessary suffering.

### 2.11. Histological Analysis

Earthworm samples subjected to tail amputation with and without GA-conjugated extract topical exposure were euthanized with -70% cold alcohol and then fixed in 4% paraformaldehyde in 0.1 M PBS, pH 7.4 at 4°C. Paraplast (Bio-Optica Spa, Milan, Italy)-embedded cells were cut into 7 *μ*m thick sections. Histological observations were performed using Masson Goldner's trichrome stain. Sections were examined using a Leica DMRB light and epifluorescence microscope, and images were acquired by optical microscopy (×100 and ×200 magnification) using a Leica DMI 4000 B microscope (Leica Microsystems GmbH, Wetzlar, Germany).

### 2.12. Gene Expression Protocol

Earthworms were rinsed with distilled water, and the last three rings were excised with a scalpel and immediately placed in TRIzol for mRNA extraction. RNA was extracted using Quick-Zol (TRIzol, Ludwig Biotech Co., Alvorada, Brazil), according to the manufacturer's instructions, and was quantified using a NanoDrop™ 1000 spectrophotometer (Thermo Fisher Scientific). Further, RNA samples were treated with 0.2 *μ*L of DNase (Invitrogen Life Technologies) at 37°C for 5 min to digest any DNA contamination and at 65°C for 10 min. Gene expression was evaluated by qPCR using a thermocycler (Axygen® 261 MaxyGene II Thermal Cycler, Corning-Life Sciences, Tewksbury, MA, USA).

RT-qPCR was performed using 19 *μ*L of a mix containing the iTaq Universal SYBR Green Supermix (Bio-Rad Laboratories) and 1 *μ*L cDNA sample. The parameters used were an initial denaturation step of 3 min at 95°C, followed by 40 cycles of 95°C for 10 s, 60°C for 30 s, and a melting step to generate a melting curve from 65°C to 95°C with an increase of 0.5°C over 5 s. PCR primers for evaluation of *SOX4* gene expression were as follows: forward, 5-′CAGGGAGTACCCGGACTACA-3′ and reverse 5-CCACGAGTCACTTACCAGCA-3′. The expression level of *β*-actin was used as an internal control. Relative expression was calculated using the comparative Ct method and was expressed as fold expression compared to the control.

### 2.13. Statistical Analysis

Comparisons among treatments were performed using GraphPad Prism software (v.8.02, 2019). Data were compared among treatments using analysis of variance (ANOVA), followed by Tukey's post hoc test or by Kruskal-Wallis nonparametric analysis of variance, followed by the Wilcoxon-Mann–Whitney post hoc test. Data in the graphs are presented as means plus 95% confidence intervals (CIs), which were the most representative statistical parameters for the comparison of treatments. Categorical variables were compared using chi-squared or Fisher's exact tests and are presented as relative frequencies (%). The area of brown bodies (BB; in *μ*m) was compared using the Digimizer image analysis software package (v.5.4.1, MedCalc Software, Belgium), which allowed manual measurements, as well as automatic object detection, for the measurement of object characteristics. All tests were considered statistically significant at *p* ≤ 0.05.

## 3. Results

### 3.1. Identification of Chemical Compounds in GA-Conjugated Extracts

An overview of all compounds identified in the GA-conjugated extracts analyzed by ESI-ToF-MS is shown in Table 1. In addition to caffeine, which is an alkaloid, polyphenols present mainly in guarana extracts were identified, such as catechin, gallic acid, quercetin, and epigallocatechin gallate (EGGC). However, other polyphenols were also observed, such as diosmetin, which is a flavone derived from luteolin, methyl gallate, and torachrysone, which belong to the class of naphthalenes. A macrocyclic peptide molecule (oscillacyclamide A) was also detected in the extract. Three molecules detected in the analysis could not be identified. The presence of citric acid was expected because this molecule was used to lower the pH in the extraction process, which gave rise to the GA-conjugated extract.

### 3.2. Indicators of Safety and Biological Capacity of GA-Conjugated Extracts

**Figure 1:**
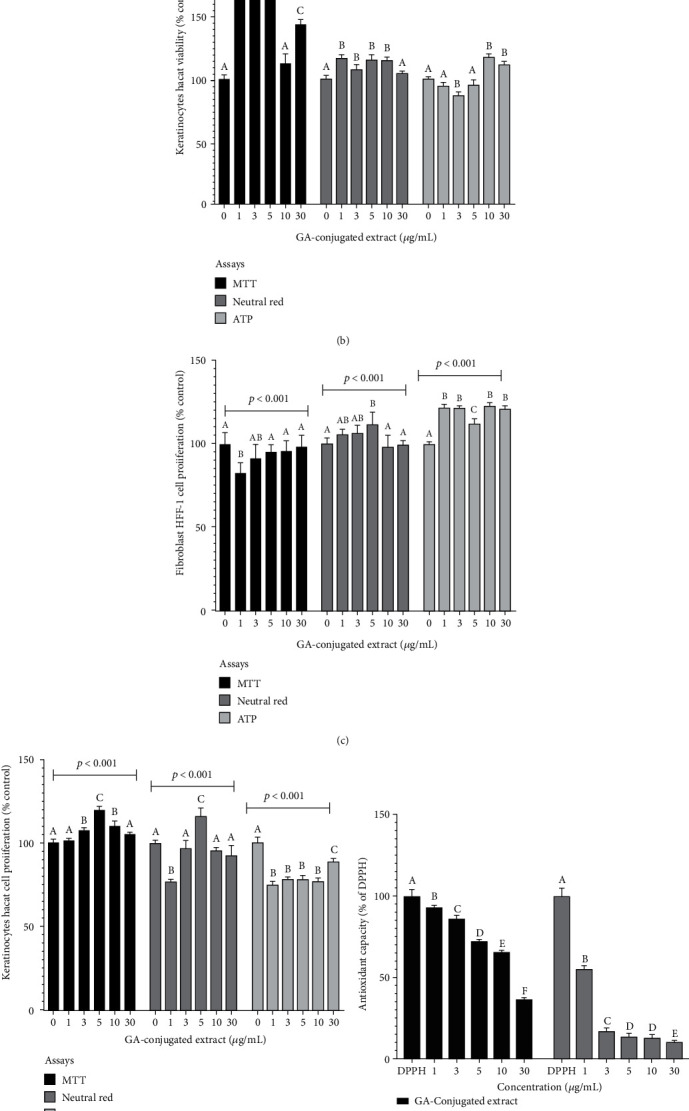
The *in vitro* effects of GA-conjugated extract at different concentrations on HFF-1 human fibroblasts and HaCaT keratinocytes cultures: (a, b) cellular viability in 24 h cell cultures; (c, d) cellular proliferation in 72 h cell cultures. Data were generated from three different protocols: MTT assay (3-[4,5-dimethylthiazol-2-yl]-2,5 diphenyl tetrazolium bromide), neutral red assay, and ATP assay. The (e) antioxidant and (f) genoprotective capacity of GA-conjugated extract at different concentrations was quantified by (e) DPPH and (f) GEMO assays, respectively. Statistical comparisons were performed by one-way analysis of variance followed by post hoc Tukey test. Different letters indicated significant differences at *p* < 0.05.

The cytotoxic effects of GA-conjugated extract on keratinocytes and fibroblasts were analyzed using three complementary assays. Data from MTT and neutral red assays showed that all extract concentrations significantly increased cell viability compared to controls in 24 h cell culture. However, the ATP assay showed an increase in the viability of fibroblasts cultured at 10 and 30 *μ*g/mL (Figures 1(a) and 1(b)). In 72 h cultures, GA conjugates triggered slightly increased cellular proliferation in MTT and neutral red assays for both fibroblasts and keratinocytes. However, an antagonistic action was observed in these cells, when considering the ATP assay. Whereas fibroblasts presented higher ATP levels than controls (Figure 1(c)), keratinocytes exhibited lower ATP concentrations than controls (Figure 1(d)). The GA-conjugated extracts showed high antioxidant capacity and genoprotective capacity in the analysis performed for DPPH and GEMO assays (Figures 1(e) and 1(d)). Overall, the results indicated that GA-conjugated extract at 5 *μ*g/mL concentration could result in biological activity in the wound healing process.

### 3.3. *In Vitro* Wound Scratch Assay

**Figure 2:**
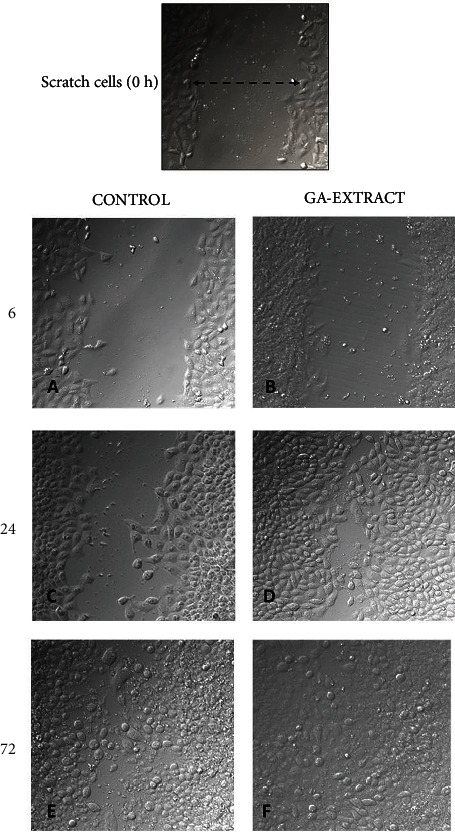
Evaluation of GA-conjugated extract at 5 *μ*g/mL concentration on HFF-1 human dermal fibroblasts submitted to scratch assay experiment. Fibroblast migration was evaluated by microphotographs (200x magnification) 6, 24, and 72 h after the cell cultures are torn with the aid of a pipette tip. To determine the cell numbers migrating during scratch closure, the perimeter of each scratch was traced and the cells inside the area of closure were counted using Digimizer software. Six wells were counted for each condition, and three independent experiments were performed, and data are presented as mean ± SD of cells that migrated inside the scratch of a single representative experiment. The migration of cultures supplemented with GA-conjugated extract in relation to nonsupplemented cultures was compared by the Student *t*-test and was significantly higher (*p* < 0.05) in those receiving the extracts than in controls at the three time points (6, 24, and 72 h) in which they were evaluated.

The GA-conjugated extract effects in *in vitro* scratched assays were employed to evaluate dermal fibroblast migration, which is a very important event for wound contraction and further cellular proliferation. Cell migration was observed at 6, 12, and 24 h after scratching (Figure 2). Cell cultures supplemented with 5 *μ*g/mL GA-conjugated extract exhibited a greater extent of migration than controls (Figures 2(a)–2(d)). Moreover, cellular proliferation observed in confluent monolayers after 72 h of cell culture was higher in fibroblast GA extract-exposed cells than in controls (Figures 2(e) and 2(f)).

### 3.4. Oxidative Metabolism Modulation in Scratched Fibroblasts

**Figure 3:**
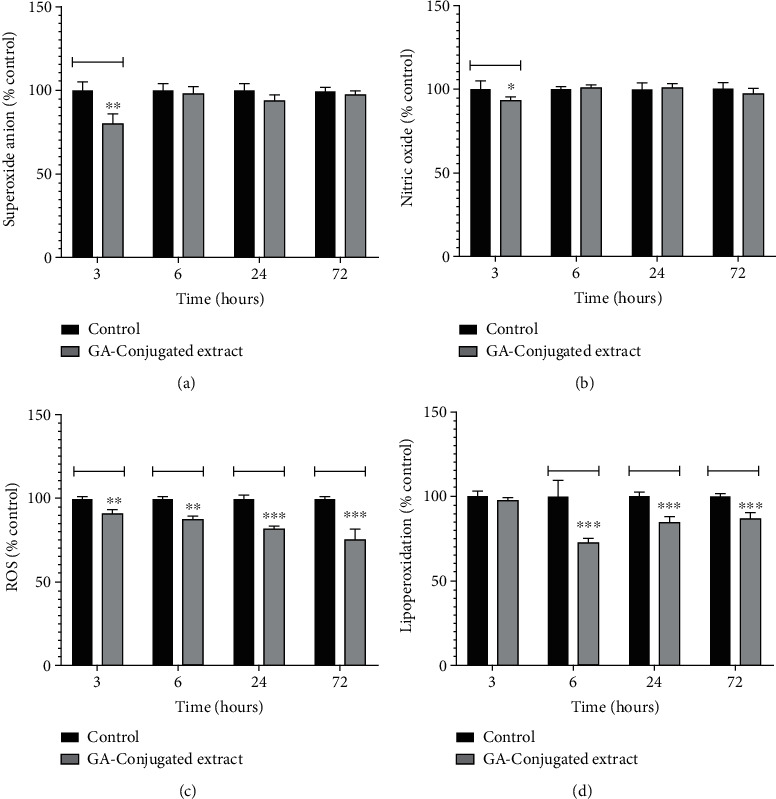
The *in vitro* modulatory effects in four oxidative metabolism markers (superoxide, nitric oxide, reactive oxygen species (ROS), and lipoperoxidation) of GA-conjugated extract at 5 *μ*g/mL concentration on 3, 6, 24, and 72 h cultures of HFF-1 human dermal fibroblasts. Data are presented as relative percentage of control. Statistical comparisons were performed by one-way analysis of variance followed by post hoc Tukey test. Different letters indicated significant differences at *p* < 0.05.

The potential role of oxidative metabolism modulation in the migration and regeneration of scratched fibroblast monolayers was analyzed after 3, 6, 24, and 72 h of cell culture. Superoxide and NO levels decreased significantly in 3 h fibroblasts cultures supplemented with GA extract compared to controls. However, the concentrations of these oxidant molecules were similar between the treatment groups (Figures 3(a) and 3(b)). In contrast, ROS levels decreased in a time-dependent manner in fibroblasts exposed to GA compared to those in controls (Figure 3(c)). The levels of lipoperoxidation measured by TBARS quantification also decreased significantly in 6, 24, and 72 h cell cultures when compared to controls.

**Figure 4:**
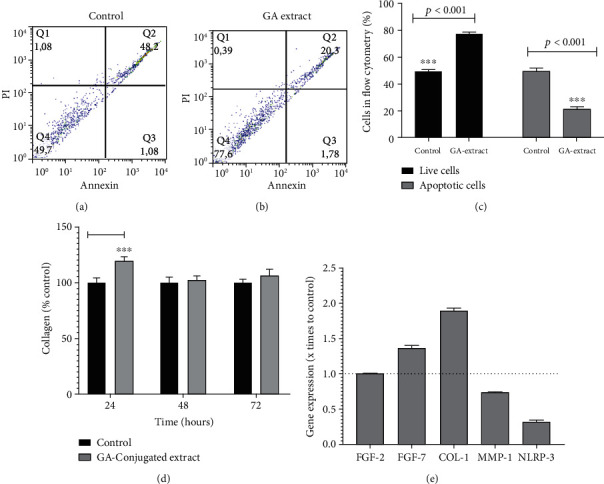
The *in vitro* modulatory effects of GA-conjugated extract at 5 *μ*g/mL concentration on HFF-1 human dermal fibroblasts by evaluation of apoptosis rate, collagen quantification, and expression of genes related to wound healing processes. (a, b) Representative flow cytometry of cell cultures in the apoptosis analysis with and without extract supplementation. (c) Comparison of dead and live cell frequency between culture extract supplemented and control by the Student *t*-test. Data are presented as relative percentage of control calculated from three replications. (d) Collagen concentration in fibroblasts 24, 48, and 72 h cell cultures compared by one-way analysis of variance followed by post hoc Tukey test. Data are presented as relative percentage of control. Different letters indicated significant differences at *p* < 0.05. (e) Gene expression: values are normalized by beta-actin 1 gene and represent their value expressed in relation to the control with reference value 1 (dashed line in the graph).

To assess the impact of GA extracts on the survival and modulation of apoptosis and necrosis of scratched cells, flow cytometric analysis in 24 h cultures was conducted. In this analysis, the frequency of live cells was significantly lower in cultures exposed to GA extract than in controls. An inverse situation was observed in relation to the frequency of cells in early and late apoptosis, which was higher in the control group than in cells exposed to GA extract. However, the frequency of necrotic cells was similar between the two groups (Figures 4(a)–4(c)).

Cells exposed to GA extract showed higher collagen concentration in 24 h cultures, which was consistent with the scratched closure pattern observed in Figure 2. However, in 48 h and 72 h cultures, collagen levels were similar between cells exposed or not exposed to GA extract (Figure 4(d)). Considering the more rapid pattern of cell confluence in cultures exposed to GA extract, analysis of its effects on the modulation of genes related to healing processes and dermal fibroblast function was also conducted. The transcriptional levels of *FGF2* were similar between the two groups. However, *FGF7* and *COL1* genes were overexpressed in cultures exposed to GA extract compared to the controls. On the other hand, downregulation was observed for the *MMP1* and *NLRP3* genes in the group exposed to the GA extract compared to the controls (Figure 4(e)).

**Figure 5:**
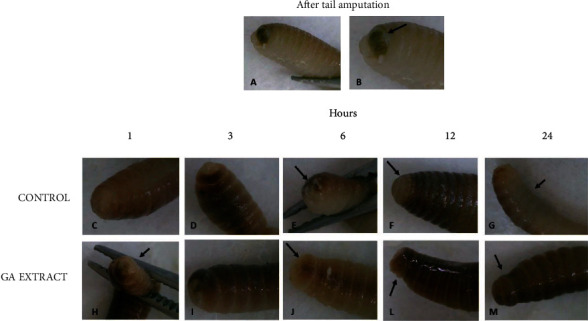
Representative photographs of the surgical incision in the anterior segments of *Eisenia fetida* earthworms topically treated with GA-conjugated extract at 5 *μ*g/mL concentration. Soon after the incision, the wound received a single topical dose of 2 *μ*L of phosphate buffer (control) or extract solution. The earthworms were then kept in Petri dishes, and the regeneration process was evaluated in different periods of time. (a, b) Right after the incision, it is possible to observe the worm's intestinal tube (in yellowish-brown color indicated by the arrow). (c, h) One hour after cutting, the entire region was covered with mucus and seems to be more contracted in earthworms that received the extract treatment than in controls. (d, i) Three hours after cutting, the contraction of the wound is already well established and it is possible to see new transparent tissue starting to form. At some point, the wound is still open. Six hours after cutting, the wound still has open parts in the control worms (e, arrow) and is completely closed with new tissue in the worm topically treated with the extract (j, arrow); 12 hours after the incision, the wound of the control worms is completely regenerated with new whitish tissue (f, arrow). In earthworms treated with the extract, the regenerated area is already pigmenting and differentiating into the segments that have been cut (l, arrow). 24 h after the incision, the entire area was regenerated. However, pigmentation in the ventral region, which is slower than the dorsal region, has not yet occurred in the control earthworms (g, arrow), while in those treated topically with the extract, pigmentation is practically complete both in the dorsal region and in the ventral region of the animal's body (m, arrow).

**Figure 6:**
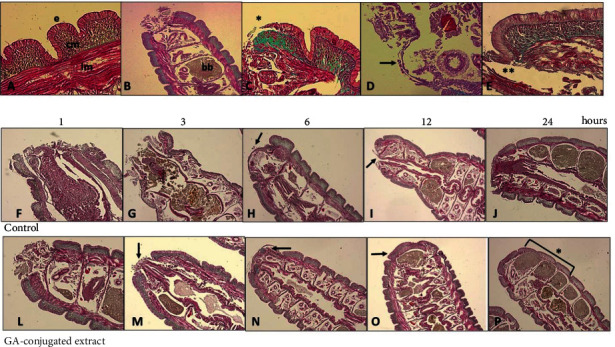
Representative histological analysis of *Eisenia fetida* earthworms submitted to posterior surgical incision of the three last segments with and without topical treatment using GA-conjugated extract at 5 *μ*g/mL concentration. The slides were stained with Masson-Goldner trichrome kit that is used for visualization of muscles, collagen fibers, connective tissues, and keratin. (a) Body wall histology showing the (e) epidermic layer that is directly connected with circular muscle layer (cm) immersed in an extracellular matrix richest in collagen. Immediately afterwards, there is the longitudinal muscular layer (lm) that is covered with the peritoneum that delimits the earthworm's coelomic cavity. (b) Posterior segments (metameres) showing the region of the surgical incision. In this place, there are spaces full of brown bodies (bb), which are structures that contain immune cells (cellomyocytes) that trapped and destroyed pathogens or linked to impurities and underwent melanization. When these cavities are completely filled, the worm performs autotomy to free them and then regenerates the posterior region. (c, d) Detail of the incision site showing migration (∗) of circular muscle cells migrating to initially close the wound. These cells behave similarly to human fibroblasts when skin damage occurs. (e) Only after migration and onset of proliferation of the smooth muscle layer does epidermis proliferation occur. (f–j) Sequence of healing processes in control earthworms at 1.3, 6, 12, and 24 hours. After this period, the region is in the final stage of regeneration of the incised segments. (l–p) Sequence of healing processes in earthworms topically treated with GA-conjugated extract showing acceleration in regenerative processes in relation to control. After 24 hours, the segments are fully regenerated (p, ∗).

The second group of experiments involved *in vivo* analyses using *E. fetida* as an experimental model in which the last three segments of the tail were surgically removed and treated topically with GA extract. We concomitantly analyzed the results of macroscopic and microscopic observations, as summarized in Figures 5 and 6.

In comparisons between the regeneration processes in controls and earthworms topically treated with GA extract, the following events were considered. Macroscopically, immediately after tail amputation, it was possible to visualize the intestines, which had a brownish color (Figures 5(a) and 5(b)). Soon after, there was a large production of mucus at the wound site (Figure 5(e)), followed by wound retraction and cell proliferation initially in a longitudinal direction, forming a more elongated structure covered by whitish tissue (Figure 5(f)). Afterwards, this tissue darkened, and the three cut segments could be visualized again (Figure 5(m)).

In histophysiological terms, it is important to highlight that in the posterior terminal region, the structure of the tissues of the earthworm body wall is very similar to that of other sites. The body wall tissue is superficially composed of a pseudostratified epithelial layer interspersed with mucous cells, which secrete a layer of chitin containing collagen and a basal lamina that is held together with the adjacent circular muscular layer. Below the circular muscle layer is a thick layer of longitudinal muscle cells with a cytohistological pattern resembling that of vertebrate smooth muscle (Figure 6(a)). Although earthworms have complete circulation, they do not have red cells, and hemoglobin is directly dissolved in the hemolymph.

Immediately after cutting, the terminal part of the intestines was exposed, followed by rapid tissue contraction and mucus production (Figures 5(b) and 6(c)). This process was visualized 1 h after tail amputation and was more rapid in GA extract-treated earthworms than in controls (Figures 5(c), 5(h), 6(f), and 6(l)). The earthworm does not have well-established connective tissue, but circular muscle cells secrete a network of collagen that supports this tissue (Figure 6(a)). When an injury occurs, hemoleucocytes present in the fluid coelom and circular muscle cells migrate rapidly to the injury site. Therefore, these latter cells operate as fibroblast-like cells, migrating to the local incision (Figures 6(a) and 6(d)). At this point, reepithelialization and production of the collagen network in the circular muscle layer begin. Concomitantly, the longitudinal muscle layer also proliferated (Figure 6(e)). The final stage of regeneration was observed when the three segments were darkened in both the dorsal and ventral regions and could be visually identified. As tail clipping involves only three segments, a large part of this regenerative process takes place within the first 24 h after incision.

Comparison of this process in the two groups of earthworms showed an acceleration of the relevant differences between the two treatments. In control earthworms, the process of migration and wound closure with these cells occurred up to 6 h after cutting (Figures 6(g) and 6(h)). However, in earthworms topically treated with GA extract, 3 h after the cut, the wound was almost completely closed, and at 6 h, it was already possible to observe the formation of multiple layers at the incision site (Figures 6(m) and 6(n)). This process can also be visually observed, as shown in Figures 5(e) and 5(j).

Twenty-four hours after the tail incision in earthworms treated with GA extract, complete and darkened segmentation was observed in the ventral and dorsal regions, whereas in control earthworms, this process was more advanced only in the dorsal region (Figures 5(g) and 5(m)). Histological analysis also confirmed that regeneration of the three segments was in the process of completion in earthworms treated with GA extract (Figures 6(j) and 6(p)). These results indicated that, on average, the regeneration rate was 6-12 h faster in earthworms treated with GA extract than in control animals.

## 4. Discussion

Based on previous evidence, the effects of a GA-conjugated extract prepared from roasted guarana seed and fresh seeds of açai berries on wound healing models were tested. The results showed that GA extract did not present cytotoxic or extensive proliferative effects on human keratinocytes and fibroblast cells in culture, showing potential antioxidant and genoprotective capacities that are relevant properties in healing or in regenerative substances of the integument. In both fibroblasts and earthworms, GA extract increased healing speed, with no cytohistological abnormalities observed during the process. Analysis of possible causal mechanisms associated with the effects of GA extract on human fibroblasts revealed antioxidant and genomodulatory activities related to the healing process.

These results will be discussed in greater detail below, starting with the chemical matrix of the GA-conjugated extract identified by ESI-ToF-MS. The GA-conjugated extract was produced by mixing roasted guarana powder seeds with fresh pulped açai seeds. The presence of caffeine is expected in guarana extracts, as this plant has high concentrations of this alkaloid [15]. The identification of polyphenols such as gallic acid, catechin, EGCG, and quercetin could also be anticipated, as many studies have described the presence of these molecules in guarana extracts, as well as in açai [18, 34, 35]. In a study conducted by [36], the authors described the presence of four flavones, luteolin and velutin, in açai.

Diosmetin, whose precursor was detected in the chemical matrix of the GA-conjugated extract, is also a flavone chemically close to luteolin. Therefore, it is possible that this molecule derives from the extraction of fresh açai seeds. All of these known molecules have antioxidant and anti-inflammatory activities, as described in the literature [37–41]. Some of these molecules appear to have healing properties, including EGCG [42] and gallic acid [43]. In addition, gallic acid, EGCG, and caffeine seem to act against complications related to healing processes, such as keloid formation [44–46].

Three other less-investigated polyphenols were also detected in the extract: the flavone diomestine, found in legumes such as *Acacia farnesiana*, and in the leaves of *Olea europaea* [47], torachrysone, a molecule belonging to the group of naphthoquinones, molecules widely distributed in plants, and cystonoside F. Previous studies have described the antioxidant, anti-inflammatory, and antimicrobial properties of these polyphenols [48–52]. In addition to these polyphenols, a macrocyclic peptide was identified, oscillacyclamide A, for which there are practically no studies regarding its biological properties. Even so, as highlighted by [53], these peptides show great promise as therapeutics because they have increased target binding affinity and selectivity, are more stable against proteolytic enzymes, and often have higher membrane permeability than their linear counterparts. Therefore, this information indicates that the chemical matrix of the GA-conjugated extract contains bioactive elements that contribute to their antioxidant and anti-inflammatory properties and to modulation of the expression of relevant genes in healing processes.

Plant extracts often have relevant therapeutic properties, but their use is restricted because of the toxicity associated with them. For this reason, we initially evaluated the potential cytotoxic and cell proliferative effects of GA-conjugated extract using keratinocytes and fibroblasts that are in direct contact when topically applied. Although there is some level of variation between the results obtained from the three different assays employed here (MTT, neutral red, and ATP), the set of results did not reveal any relevant toxic effects. These results are in accord with those of previous studies that did not indicate toxicity associated with the supplementation of cell cultures with guarana or açai extract [20, 34].

Two tests were conducted to determine the biological capacity of the extracts. The first evaluation of the scavenging power of the DPHH radical by the GA-conjugated extract showed that it has a relevant antioxidant capacity. The second trial evaluated the extent to which the extract was able to decrease the rate of degradation of dsDNA molecules exposed to high concentrations of H_2_O_2_. The results also suggested that the GA-conjugated extract exerted a genoprotective capacity. In the GEMO test, described by [28], isolated dsDNA is exposed to H_2_O_2_, which causes extensive strand breaks in the DNA backbone. Since the PicoGreen dye has high affinity for dsDNA but does not bind to nucleotides and single-stranded DNA, these breaks reduce the fluorescence of the reaction. However, the presence of GA extract decreased DNA breakage rates, indicating its genoprotective activity. These results allowed the choice of the GA extract concentration to be 5 *μ*g/mL, which was later used in complementary tests *in vitro* with fibroblasts and *in vivo* with earthworms.

The results showed that, in both fibroblasts and earthworms, GA extract induced a greater speed in the wound healing process. Macroscopic and histological analyses of surgical incisions of earthworm tails revealed accelerated healing processes, corroborating the results observed in human fibroblasts. We highlight here that the use of earthworms as an experimental model for regeneration studies has been gaining popularity for a number of reasons. First, earthworms have long been used in ecotoxicity studies to assess soil quality in relation to environmental pollutants. This more primitive organism also has great regenerative capacity while having some cytofunctional elements that have certain similarities to the human immune system [10]. Furthermore, earthworm husbandry involves low maintenance cost and incurs fewer ethical problems than the use of vertebrates in experiments involving surgical incision. Although the data using earthworms provided evidence of the role of GA extract in wound healing in *E. fetida*, analyses of potential causal mechanisms were performed only in fibroblasts. This is because *E. fetida* is still a relatively new model in studies of regeneration involving environmental variables, such as plant extracts. Consequently, it will be necessary to establish future analyses of biochemical and molecular markers related to the role of inflammation in healing and other metabolic pathways.

Therefore, the potential effect of GA extracts on oxidative metabolism was investigated in fibroblasts. The results showed a generalized antioxidant effect when the levels of total ROS were evaluated. These values remained lower than in control cells at all time points analyzed. The lipoperoxidation rate was also lower in 6 h fibroblast cultures subjected to the scratch assay. Two other oxidative markers that are directly associated with inflammatory responses (superoxide and NO) were also analyzed. The results showed a decrease in the levels of these two oxidative molecules in 3 h cultures. Thereafter, superoxide and NO levels remained similar to those in control cells. In addition, scratched cultures supplemented with GA extract exhibited lower apoptosis rates than controls.

Previous investigations such as the study performed by [54] showed that, in the presence of prosenescence molecules such as advanced glycation end products (AGEs), dermal fibroblasts are induced to undergo apoptosis, presenting an increase in ROS and overexpression of the *NRLP3* gene, which is directly associated with inflammasome formation. Although this study did not expose dermal fibroblasts to molecules such as AGEs, supplementation of cultures with GA extract showed the opposite effect. That is, 24 h cultures exposed to GA extract showed lower levels of ROS and downregulation of the proinflammatory gene *NRLP3*. It is possible that the peak oxidative stress and proinflammatory responses caused by scratching decreased in cultures treated with GA extract. This is because there is evidence that the triggering of initial oxy-inflammatory processes is relevant for wound healing to occur [55].

Other relevant results concern the induction of greater collagen formation, *Col-1* gene overexpression, and faster scratch closure in fibroblast cultures and those supplemented with GA extract. Moreover, differential modulation of other genes related to tissue regeneration and dermal function was observed in fibroblast cultures supplemented with GA extracts. This is the case with *FGF7* overexpression in fibroblast GA extract supplements, which is considered a relevant gene for organogenesis and mediation of wound healing in mammals [56]. In addition to downregulated *NRPL3* expression, this result was also observed for *MMP1*. Collagenases such as MMP-1 play a major role in the healing process. When a physical lesion occurs, generating a wound, MMP-1 is activated assisting the healing process by elimination of damaged proteins, destroying the provisional extracellular matrix, facilitating fibroblast migration, and remodulation of granulation tissue [57]. Again, it is possible that the low expression of *MMP1* observed in 24 h cell cultures may be related to the fact that the GA extract accelerated the wound healing process compared to control cultures.

Since *in vitro* assays have some limitations in assessing the properties of certain extracts or drugs, the results described here from fibroblast cell culture were corroborated by *in vivo* assays involving *E. fetida*. In summary, despite limitations related to *in vitro* and *in vivo* studies, the results obtained from this translational investigation suggest that GA extract has a potential therapeutic effect on the healing of acute wounds, such as those that occur during surgical interventions.

## Figures and Tables

**Table 1:** Identification of the chemical compounds from acai-guarana extract by ESI-ToF-MS.

No.	Observed, *m*/*z*	Possible molecular structure	Error, ppm	MS/MS fragmentation (collision energy)	Elucidation	References
*Positive ionization mode, [M-H]+*						
1	381.0804	C_14_H_9_N_10_O_4_ C_13_H_13_N_6_O_8_ C_17_H_17_O_10_	-1.0 +2.4 -4.7	219.0287; 201.0166 (20)	4-CDOA (derivate from caffeic acid)	doi:10.1002/rcm.4662 doi:10.1016/j.pharmthera.2018.05.006
2	365.1067	C_10_H_17_N_6_O_9_ C_10_H_17_N_6_O_9_ C_27_H_13_N_2_ C_12_H_9_N_14_O C_14_H_21_O_11_	-0.8 +2.7 -3.3 -4.7 -4.7	203.0552; 185.0441 (20)	The *m*/*z* 365.1067 is a possible precursor ion of aconitate D or aconitate F (*m*/*z* 203.0552), and the *m*/*z* 185.0441 is associated to methyl gallate	doi:10.3390/md15120374 doi:10.1016/j.foodchem.2014.06.011
3	295.1854	C_9_H_19_N_12_ C_8_H_23_N_8_O_4_	-0.7 +4.1	256.0335; 148.0975 (9)	The *m*/*z* 295.1854 is a possible precursor ion of diosmetin (*m*/*z* 256.0335), and the *m*/*z* 148.0975 is associated to *d*-fagomine	doi:10.3389/fphar.2020.00824 doi:10.1007/s00216-011-5639-2
4	215.0175	C_4_H_3_N_6_O_5_	+4.7	166.0320 (13)	The *m*/*z* 215.0175 and 166.0320 are possible sequential fragments from oscillacyclamide A	doi:10.1039/c7cc05913b
5	203.0527	C_4_H_7_N_6_O_4_	-1.0	172.0115; 160.0625; 136.0645 (10)	Unknown	Unknown
6	195.0883	C_8_H_11_N_4_O_2_	+0.5	138.0671; 110.0730 (16)	Caffeine	doi:10.1021/es047985v
7	170.0785	C_7_H_6_O_5_ C_3_H_12_N_3_O_5_	-2.9 +4.7	149.0327; 128.5182 (3)	Gallic acid	doi:10.1016/j.chmed.2020.06.002
*Negative ionization mode, [M-H]-*						
8	458.9561	C_22_H_18_O_11_ C_17_H_3_N_2_O_14_	+3.7 -5.0	190.9990 (11)	Epigallocatechin gallate	doi:10.1002/rcm.1135
9	391.0296	C_14_H_3_N_10_O_5_ C_18_H_7_N_4_O_7_	+2.0 -4.9	216.9986; 191.0019; 172.9928; 110.9950 (8)	The *m*/*z* 391.0296 is a possible precursor ion of citric acid (*m*/*z* 191.0019)	doi:10.1016/j.foodchem.2017.03.050
10	341.1085	C_12_H_21_O_11_ C_10_H_9_N_14_O C_25_H_13_N_2_ C_13_H_17_N_4_O_7_ C_9_H_2_N_10_O_5_	+0.3 +0.3 +1.8 -3.5 +4.4	179.0456; 161.0357; 89.0168 (18)	Cistanoside F (caffeic acid hexosidedeoxyhexoside)	doi:10.3390/foods8100432
11	333.0244	C_15_H_9_O_9_	-0.9	Reduced signal for MS/MS analysis	Unknown	Unknown
12	289.0689	C_15_H_14_O_6_	-1.8	245.0700 (13)	Catechin	doi:10.1002/bmc.4807
13	245.0804	C_14_H_13_O_4_	-4.1	Reduced signal for MS/MS analysis	Torachrysone	doi:10.1039/C4AY02506G
14	228.9726	Unknown	—	Reduced signal for MS/MS analysis	Unknown	Unknown
15	191.0192	C_6_H_7_O_7_	0.0	111.0062 (16)	Citric acid	doi:10.1016/j.foodchem.2017.03.050
16	173.0069	Unknown	—	Reduced signal for MS/MS analysis	The *m*/*z* 173.0069 is a possible sequential fragment from quercetin	doi:10.1016/j.bjp.2016.03.009

## Data Availability

The data will be made available if the reviewers and/or editors of this journal so request.
